# DNMT1 is a negative regulator of osteogenesis

**DOI:** 10.1242/bio.058534

**Published:** 2022-03-03

**Authors:** Chen Tao, Jia Liu, Ziqi Li, Pinglin Lai, Sheng Zhang, Jiankun Qu, Yujin Tang, Anling Liu, Zhipeng Zou, Xiaochun Bai, Jianwei Li

**Affiliations:** 1 State Key Laboratory of Organ Failure Research, Department of Cell Biology, School of Basic Medical Sciences, Southern Medical University, Guangzhou 510515, China; 2 Department of Orthopedics, Affliated hospital of Youjiang Medical University for Nationalities, Baise, Guangxi 533000, China; 3 Guangdong Provincial Key Laboratory of Bone and Joint Degeneration Diseases, Academy of Orthopedics, Guangdong Province, The Third Affiliated Hospital of Southern Medical University, Guangzhou 510630, China; 4 Department of Surgery, Tan Cheng County Maternal and Child Health Care Hospital, Linyi, Shandong 276100, China; 5 Division of Orthopaedics and Traumatology, Department of Orthopaedics, Nanfang Hospital, Southern Medical University, Guangzhou 510515, China

**Keywords:** DNMT1, MSC, Osteoblast, Methylation

## Abstract

The role and underlying mechanisms of DNA methylation in osteogenesis/chondrogenesis remain poorly understood. We here reveal DNA methyltransferase 1 (DNMT1), which is responsible for copying DNA methylation onto the newly synthesized DNA strand after DNA replication, is overexpressed in sponge bone of people and mice with senile osteoporosis and required for suppression of osteoblast (OB) differentiation of mesenchymal stem cells (MSCs) and osteoprogenitors. Depletion of DNMT1 results in demethylation at the promoters of key osteogenic genes such as RORA and Fgfr2, and consequent upregulation of their transcription *in vitro*. Mechanistically, DNMT1 binds exactly to the promoters of these genes and are responsible for their 5-mc methylation. Conversely, simultaneous depletion of RORA or Fgfr2 blunts the effects of DNMT1 silencing on OB differentiation, suggesting RORA or Fgfr2 may be crucial for modulating osteogenic differentiation downstream of DNMT1. Collectively, these results reveal DNMT1 as a key repressor of OB differentiation and bone formation while providing us a new rationale for specific inhibition of DNMT1 as a potential therapeutic strategy to treat age-related bone loss.

## Abstract (precis)

**Summary:** DNMT1 is overexpressed in sponge bone of people and mice with senile osteoporosis and required for suppression of osteoblast (OB) differentiation of mesenchymal stem cells (MSCs) and osteoprogenitors.

## INTRODUCTION

Bone-derived mesenchymal stem cells (BMSCs) are the multipotent progenitors responsible for maintaining the non-hematopoietic cell populations of the bone marrow, cortical bones (Fernandez-Moure et al., 2015), and trabeculae (Wang et al., 2015; Sottile et al., 2002). The lineage-specific commitment of BMSCs to osteoblasts (OBs), chondrocytes, or adipocytes and consequent formation of bone, cartilage, or marrow fat, respectively, is a tightly regulated process that is dependent on various signaling mechanisms (Pierce et al., 2019). The commitment into OBs, chondrocytes or adipocytes in the BMSCs population can be coordinately modulated by Wnt, transforming growth factor-beta (TGFβ)/bone morphogenetic proteins (BMPs), hedgehog proteins, Notch, endocrine hormones such as parathyroid hormone (PTH), and various growth factors through the coordination of master OB transcription factors (TFs) β-catenin/TCF4, runt-related transcription factor 2 (Runx2) and Osterix 1 (Osx1/Sp7), chondrogenic TFs Sox9/Sox6/Sox5 and adipogenic TFs peroxisome proliferator-activated receptor γ (Pparγ) and CCAAT/enhancer-binding protein α (c/EBPα) (Pierce et al., 2019; Chen et al., 2016; Kawakami et al., 2006; Akiyama and Lefebvre, 2011). Despite the fact that the roles of these key programs in osteogenesis, chondrogenesis, or adipogenesis have been revealed, the epigenetic mechanism governing the balance of them is largely unknown.

Most of the CpGs dinucleotides in the mammalian genome are found in clusters that form so-called CpG islands, often at gene promoters. CpGs are the primary targets of DNA methylation to generate 5-methylcytosine (5mC) in mammalian cells. 5mC is enzymatically deposited and removed by DNA methyl transferases (DNMTs) and demethylases of the ten-eleven translocation (TET) family, respectively (Schubeler, 2015). DNMT1 was the first identified and is the most abundant DNMT type in mammalian somatic cells (Goll and Bestor, 2005; Bestor, 2000) responsible for copying DNA methylation onto the daughter DNA strand after DNA replication while DNMT3A and DNMT3B, together with DNMT3L, catalyze *de novo* methylation in non-proliferating cells (Schubeler, 2015). The functional relevance of DNMT1 during mammalian embryo development is highlighted by evidences that ablation of Dnmt1 in mice results in embryonic lethality by day 8.5 post coitum (Li et al., 1992). DNMT1 is essential to maintain both stem cells (Sheaffer et al., 2014) and progenitors in intestinal epithelium (Elliott et al., 2015), to maintain progenitor cell survival during pancreatic organogenesis (Georgia et al., 2013), as well as to restrain alveolar type 2 cell fate in lung endoderm development (Liberti et al., 2019). Despite the role of DNA methylation has been implicated in cartilage development (Yamashita et al., 2018; Zhang et al., 2016; Taylor et al., 2016), the precise contribution of DNMT1 in bone development and degeneration is currently unclear.

In the present study, we show that DNMT1 protein expression is reduced during OB differentiation, while enhanced in osteoprogenitors and chondroblasts of trabecular bone from human and mice with senile osteoporosis (SOP). Depletion of DNMT1 promoted differentiation of both OBs and chondrocytes *in vitro*. Remarkably, DNMT1 depletion rendered hypomethylation at nearly all chromosomes, especially promoter CGIs of genes involved in ossification, bone morphogenesis, and chondrocyte differentiation, including but not limited to Fgfr2, RORA, and Itga8, while suppressing their expression. Importantly, the current study revealed a novel role of DNMT1 in control of chondrocyte and OB differentiation of MSC and consequent skeletal development, implicating a potential role of DNMT1 in the development of osteoporosis. Thus, an in-depth investigation of the effect of targeted DNMT1 inhibition on the treatment of osteoporosis is warranted in light of our findings.

## RESULTS

### DNMT1 expression is enhanced in trabecular bone of aging human and mice

**Fig. 1.**
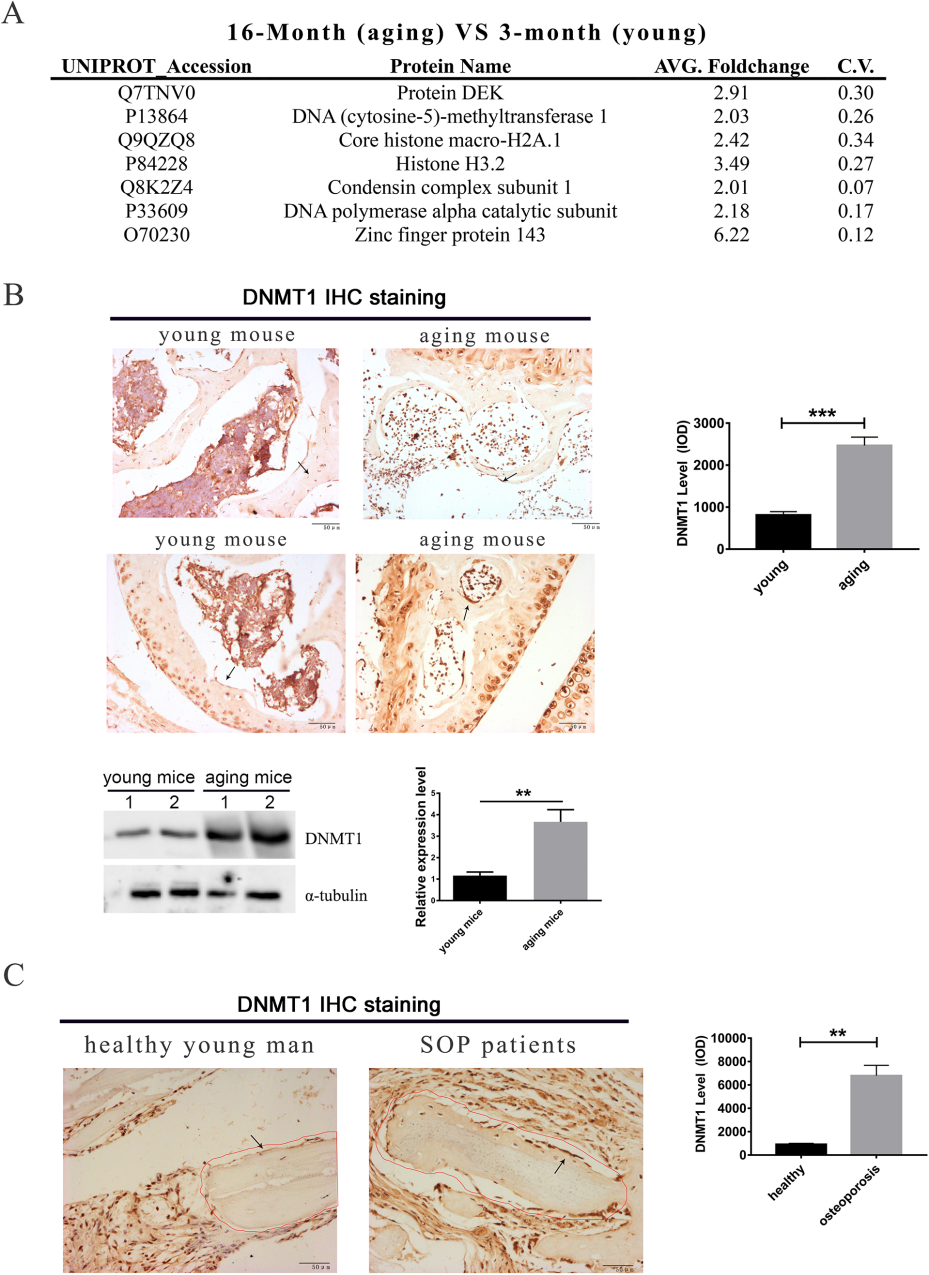
**DNMT1 expression is enhanced in trabecular bone of aging human and mice.** (A) iTRAQ-LC-MS/MS proteomic assay of the distal femur lysate of the 3-month (control) and 16-month (aging) C57BL/6J mice. Proteins with at least twofold upregulation were subjected to a GO_MF enrichment assay using the DAVID online software. Proteins in the ‘chromatin binding’ category were shown. (B) Upper panel, IHC staining for DNMT1 in the sponge bone or articular cartilage regions of distal femur from these mice. Lower panel, distal femora of these mice were analyzed for the indicated proteins by western blotting. Representative images were shown in the left and quantitation by Image J software was shown in the right. Representative bone lining preosteoblasts were indicated by black arrows. **, *P*<0.01, ***, *P*<0.001 (two-tailed paired *t*-tests, *n*=6). (C) IHC staining for DNMT1 in femur neck sections of healthy young males or patients with age-related osteoporosis (senile osteoporosis, SOP). Representative images were shown in the left and quantitation was shown in the right. **, *P*<0.01 (two-tailed unpaired *t*-tests, *n*=6 for healthy young males and *n*=10 for osteoporosis patients).

To get a better understanding of the regulatory mechanism of age-related osteoporosis, the differentially expressed proteins in the distal femur of 16-month C57BL/6 mice (aging mice) versus that of their 3-month-old counterparts (young mice) were analyzed by an iTRAQ-LC-MS/MS proteomic approach. Proteins upregulated in the aging mice (more than twofold) were analyzed by the DAVID Tools (https://david.ncifcrf.gov/), with proteins related to chromatin binding shown (Fig. 1A). Interestingly, DNMT1 expression was significantly enhanced in the trabecular bone and articular cartilage of aging mice (Fig. 1B). Consistently, immunohistochemistry (IHC) analysis also confirmed that DNMT1 expression level was upregulated in both the chondrocyte progenitors near the articular surface and the osteoprogenitors lining the trabecular surface in aging mice (Fig. 1B), which are demonstrated to be multipotent (Sottile et al., 2002). Likewise, an elevated DNMT1 protein level was also observed in osteoprogenitors lining the trabeculae surface in bone sections from the femoral trochanter of osteoporosis patients, in comparison with that of a healthy young male (Fig. 1C).

### DNMT1 suppresses osteogenesis *in vitro*

**Fig. 2.**
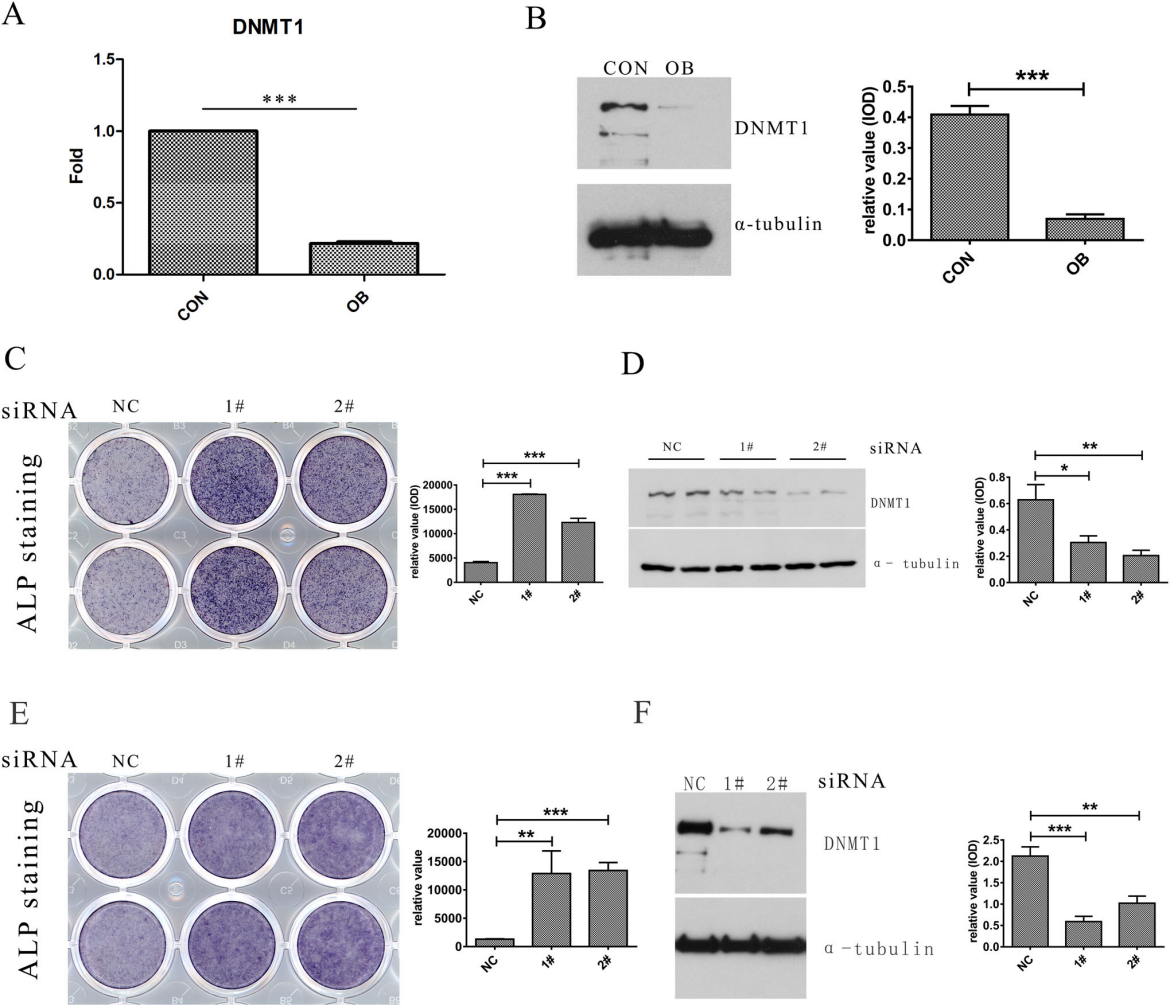
**DNMT1 suppresses osteogenesis *in vitro*.** C3H10T1/2 MSCs were induced for osteoblast (OB) differentiation with the osteogenic medium for 3 days. The cells were then harvested and lysed for either RT-qPCR (A) or western blotting (B) analysis for DNMT1 expression. C3H10T1/2 cells were transfected with NC or DNMT1 siRNA and cultured in osteogenic medium for 8 days, with OB differentiation analyzed by alkaline phosphatase (ALP) staining (C) and DNMT1 depletion examined by western blotting (D). MC3T3-E1 cells were treated as above, followed by OB differentiation analyzed by ALP staining (E) and DNMT1 depletion examined by western blotting (F). Representative images were shown in the left and quantitation by Image J software was shown in the right. *, *P*<0.05; **, *P*<0.01; ***, *P*<0.001 (two-tailed unpaired *t*-tests, *n*=3).

The mouse C3H10T1/2 mesenchymal stem cells (MSCs), which derive from C3H mouse embryos, bear characteristics suitable for studies on the stem cell commitment program (Bowers et al., 2006; Katagiri et al., 1990). C3H10T1/2 cells during OB differentiation demonstrated a marked reduction in DNMT1 expression, as manifested by both RT-qPCR (Fig. 2A) and western blotting (WB) assay (Fig. 2B). Alkaline phosphatase (ALP) is expressed by early-stage OBs and hence used as a hallmark for early OB differentiation (Gordon et al., 2010). DNMT1 depletion by siRNA resulted in a significant enhancement in OB differentiation of the MSCs, as visualized by ALP staining (Fig. 2C). The efficiency of DNMT1 knockdown and its effects on OB markers were also confirmed by WB assay (Fig. 2D). MC3T3-E1 cells represent progenitor cells committed to, but not terminally differentiated osteoblasts. Consistently, DNMT1 depletion also led to a marked enhancement in OB differentiation from MC3T3-E1 osteoprogenitors, as observed from both ALP staining (Fig. 2E) and WB assay (Fig. 2F). These results collectively suggest DNMT1 may suppress OB differentiation both at the lineage commitment step and at the later differentiation step.

### DNMT1 controls methylation of osteogenic and chondrogenic genes and suppresses their expression

**Fig. 3.**
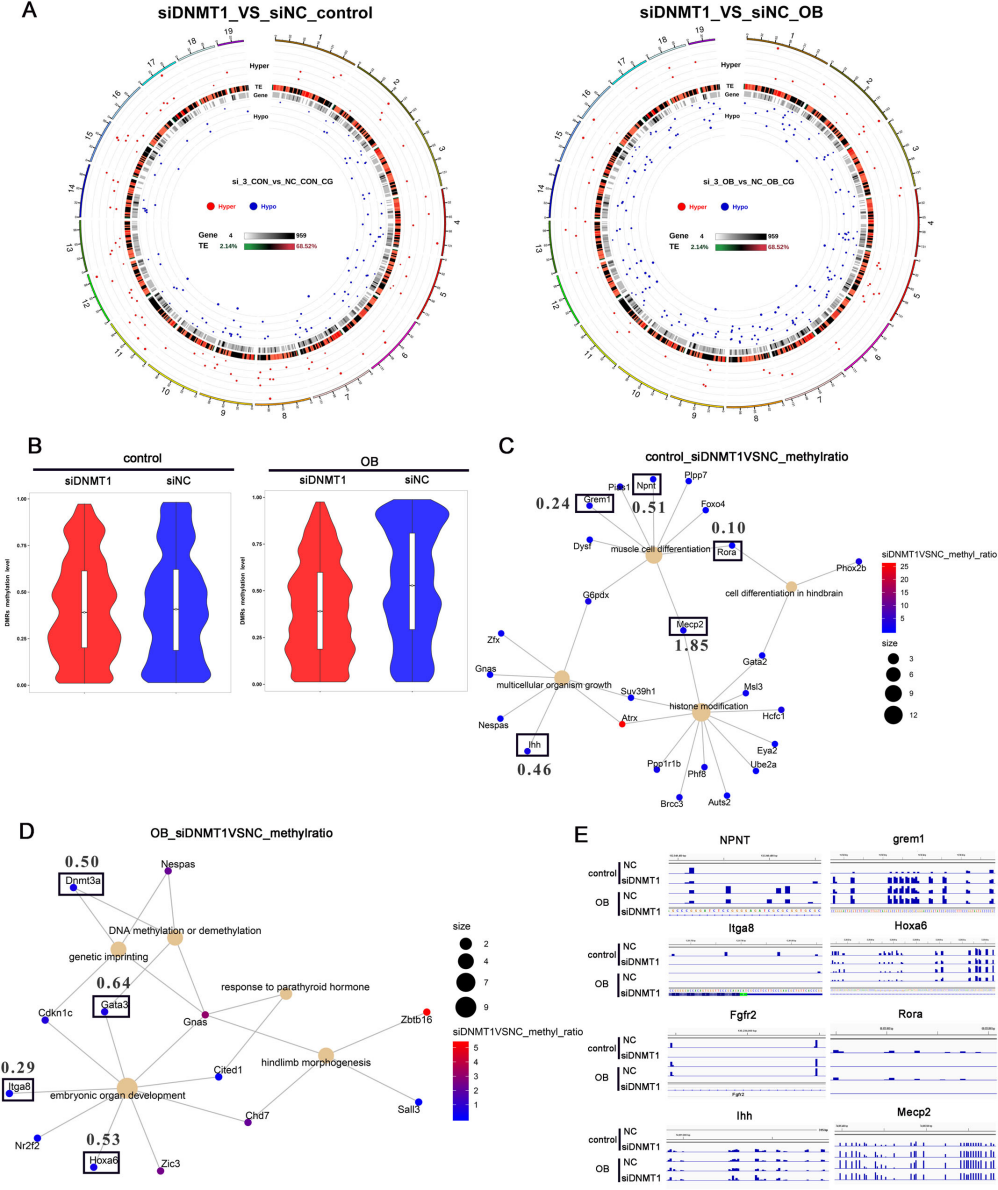
**DNMT1 controls the methylation of key osteogenic or chondrogenic genes.** C3H10T1/2 mesenchymal stem cells were transfected with DNMT1 (or non-targeting, NC) siRNA, induced for early osteoblast (OB) differentiation, and analyzed for global DNA methylation by reduced representation bisulfite sequencing. (A) A circos plot indicating the hypermethylated (red) or hypomethylated (blue) regions over all the chromosomes. Control, left panel; OB, right panel. (B) Violin plot showing the region methylation levels of these cells. The DNMT1 depleted cells were left untreated (C) or induced for early OB differentiation (D), with differentially methylated genes analyzed by a GO_BP assay and shown by a CNET plot. The most important genes were highlighted by black rectangles. The relative methylation levels (siDNMT1 VS NC) were also indicated. (E) The methylation levels and regions of these genes were viewed by IGV.

Aiming to identify potential target genes methylated by DNMT1, we performed reduced representation bisulfite sequencing (RRBS), an approach believed to cover a large majority of CpG islands at a single-nucleotide level (Nagarajan et al., 2014), to analyze the differentially methylated regions (DMRs) or loci (DMLs) in DNMT1-depleted C3H10T1/2 MSCs before or after OB differentiation for 3 days. As expected, DNMT1 depletion resulted in more hypomethylated (relative to hypermethylated) regions at the whole-genome level (Fig. 3A, left), as manifested by a circos plot. This disparity was even more prominent after early OB differentiation (3d) (Fig. 3A, right). In particular, top-methylated regions in these MSCs were slightly reduced by DNMT1 depletion at the rest state, and to a greater extent after early OB differentiation, observed from a violin plot (Fig. 3B). Gene ontology (GO) enrichment analysis for genes with differentially methylated promoter regions revealed that functional categories related to OB differentiation or organ development were enriched before (Fig. 3C) or after early OB differentiation (Fig. 3D). The relative methylation levels and locations at the promoter regions of the most representative genes were shown (Fig. 3E), including Indian hedgehog protein (IHH), Grem1, Rora, Mecp2, and Npnt before OB differentiation and Itga8, Fgfr2, and Dnmt3a after early OB differentiation.

**Fig. 4.**
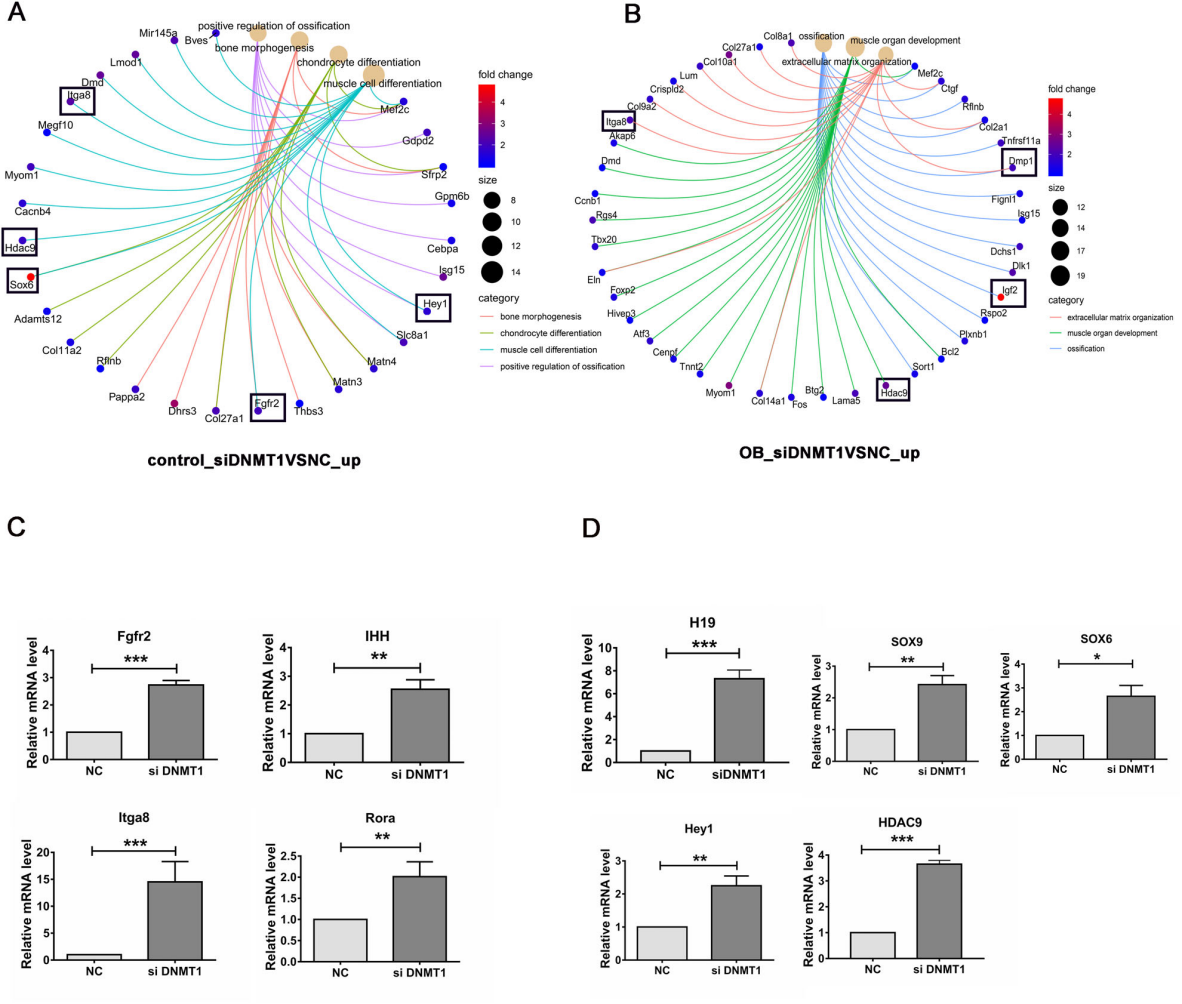
**DNMT1 controls the expression of key osteogenic or chondrogenic genes.** C3H10T1/2 mesenchymal stem cells were transfected with DNMT1 (or non-targeting, NC) siRNA, induced for early osteoblast (OB) differentiation, and analyzed for global mRNA expression by next-generation RNA-sequencing. The upregulated genes in DNMT depleted cells either uninduced (A) or during OB differentiation (B) were subjected to a GO_BP assay, with enriched GO terms and genes shown by a circular CNET plot. Fold changes are indicated with different colors, representing the log2-transformed siDNMT1/NC ratio. The most important genes are highlighted by black rectangles. Expression of the genes with (C) or without concomitant alteration (D) in promoter methylation were verified by RT-qPCR. *, *P*<0.05; **, *P*<0.01, ***, *P*<0.001 (two-tailed unpaired *t*-tests, *n*=3).

Additionally, to further clarify the downstream effects of these hypomethylated regions or loci on global gene expression, mRNA expression profile of C3H10T12 MSCs with or without DNMT1 depletion were analyzed by next-generation RNA-sequencing. Consistent with previous identified roles of DNMT1 in suppressing both OBs and chondrocyte differentiation, DNMT1 depletion resulted in enhanced expression of genes involved in positive regulation of ossification, cartilage development, bone morphogenesis, and muscle cell differentiation before OB differentiation (Fig. 4A), while upregulated expression of genes involved in ossification, muscle organ development, and extracellular matrix organization after early OB differentiation (Fig. 4B).

The upregulation of genes with concomitant promoter hypomethylation were confirmed by RT-qPCR. Consistent with RNA-seq data, DNMT1 depletion resulted in enhanced Itga8, Rora, Ihh, and Fgfr2 mRNA expression (Fig. 4C). Remarkably, the transcription of other key osteogenic or chondrogenic factors, including, but not limited to, lncRNA H19, HDAC9, Hey1, Sox9, and Sox6 (Fig. 4D) were also significantly enhanced upon DNMT1 depletion, corroborating the suppression of DNMT1 on osteogenesis or chondrogenesis. Interestingly, these genes have no detectable methylation alterations at either their promoters or gene bodies (data not shown), suggesting that their expression may not be directly regulated by DNMT1-mediated DNA methylation.

### RORA and Fgfr2 is required to suppress osteogenic and chondrogenic differentiation downstream of DNMT1

**Fig. 5.**
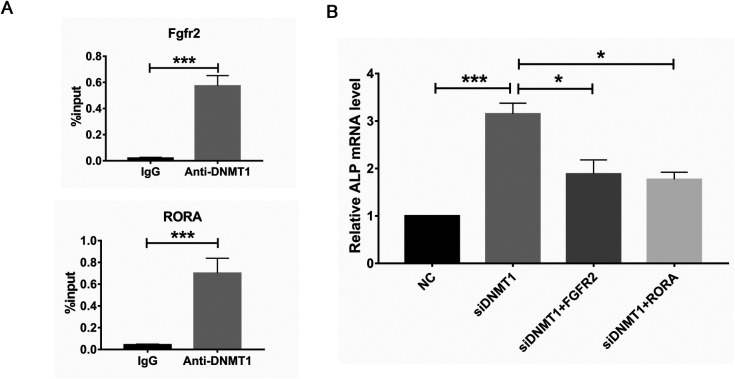
**RORA and Fgfr2 acts downstream of DNMT1 to regulate osteoblast differentiation.** (A) Binding of DNMT1 to the promoter region of RORA or Fgfr2 in C3H10T1/2 mesenchymal stem cells (MSCs) was analyzed by chromatin IP-qPCR. (B) C3H10T1/2 MSCs were simultaneously transfected with DNMT1 (or non-targeting, NC) and RORA (Fgfr2) siRNA, induced for osteoblast differentiation for 8 days, followed by examination of ALP expression via RT-qPCR. *, *P*<0.05; **, *P*<0.01; ***, *P*<0.001 (two-tailed unpaired *t*-tests, *n*=3).

Chromatin immunoprecipitation (chIP)-qPCR was performed to analyze whether DNMT1 binds to the promoter region of RORA and Fgfr2, to identify whether they are directly targeted and methylated by DNMT1. As expected, DNMT1 binds exactly to the hypomethylated region, triggered by DNMT1 depletion at the promoter of RORA and Fgfr2 (Fig. 5A), indicating that these CGIs are directly methylated by DNMT1. Consistently, simultaneous knockdown of RORA or Fgfr2 with DNMT1 markedly suppressed OB differentiation enhanced by DNMT1 depletion (Fig. 5B), suggesting that RORA and Fgfr2 are required for modulating the suppressing effect of DNMT1 on OB differentiation.

## DISCUSSION

DNMT1 has been intensively shown to promote tumorigenesis (Wong, 2020a; 2020b), while its role in mesenchyme development remains elusive. Although DNMT1 has been shown to be essential for expression of myogenic genes, myotubes formation, and consequent myogenic differentiation (Liu et al., 2016), the direct targets of DNMT1 in MSC lineage switch are largely unknown. In the current study, we show that DNMT1 expression is reduced during OB differentiation of MSCs or osteoprogenitors, but enhanced in osteoprogenitors of humans and mice with age-related bone loss. Consistently, DNMT1 depletion promotes OB differentiation of both MSCs and osteoprogenitors. In addition, by assessing global methylation alterations in CpG dinucleotides in the CpG islands as well as the resultant alterations in gene expression in response to DNMT1 depletion with a coupled RRBS-RNA-seq approach in MSCs, we demonstrated RORA and Fgfr2 is hypomethylated at their promoter regions with corresponding elevated mRNA levels downstream of DNMT1 silencing to promote MSC osteogenic differentiation. Additionally, expression of a large portion of other osteogenic or chondrogenic factors are enhanced, which may be a secondary effect of DNMT1 silencing, but also functions to amplify the osteogenic or chondrogenic signaling.

After DNMT1 depletion, key OB lineage-determining genes, such as Rora, Fgfr2, and Ihh, exhibited promoter demethylation and concomitant upregulation in their mRNA expression, suggesting that DNMT1 may function to suppress early osteogenesis. However, the expression of a large body of genes involved in osteogenesis or chondrogenesis was enhanced without prominent alteration in promoter methylation, such as Hey1, Sox9, Sox6, and lncRNA H19. The observed limited consistency between increased transcripts and demethylation in their coding genes may reflect either transcriptional alteration secondary to DNMT1 depletion, or the non-catalytic functions of DNMT1 documented previously (Espada, 2012). Interestingly, our results, together with our previous finding that overexpression of DNMT1 leads to hypermethylation of H19 promoter and consequent impairment of osteogenesis *in vitro* (Li et al., 2018), support a possible role of DNMT1-H19 signaling in OB differentiation.

Besides the hypomethylated regions, DNMT1 silencing also resulted in individual hypomethylated CpG loci at the promoter of some genes, such as *Wnt3a* and *HDAC9* (Fig. S1), with concomitant upregulation of their mRNA levels. Although most current studies have focused on the regulatory role of differentially methylated regions on gene expression, single-CpG-site based methylation difference may also significantly affect the expression of various genes (Furst et al., 2012; Lim et al., 2018; Kallenberger et al., 2019). Since HDAC9 represses adipogenic differentiation via deactivation of the C/EBPα promoter (Chatterjee et al., 2011), the hypomethylation of a single CpG loci at the promoter of HDAC9 may also contribute to its transcriptional activation and consequent repression of adipogenesis. Furthermore, we revealed that promoter of DNMT3A is methylated by DNMT1, which may explain why some CGIs became hypermethylated in response to DNMT1 silencing and implicate a potential role of this DNMT1–DNMT3A crosstalk in osteogenesis or chondrogenesis.

Taken together, using coupled analysis of both methylation profile and transcriptome regulated by DNMT1, our results suggest that DNMT1 may inhibit both OB and chondrocyte differentiation via relatively complex and non-linear mechanisms. Indeed, our finding that DNMT1 is overexpressed in the trabecular bone of patients and mice with SOP and our previous finding that DNMT1 expression is upregulated in the femoral tissues of rats with disuse osteoporosis (Li et al., 2018) together support the crucial role of DNMT1 in suppression of bone formation *in vivo*. Further studies are required to investigate the anti-osteogenic and anti-chondrogenic mechanism of DNMT1 in detail, and to evaluate the effectiveness of DNMT1-specific inhibitors, such as MG98 (Amato et al., 2012), in the prevention and treatment of age-related bone loss in animal models.

## MATERIALS AND METHODS

### Chemicals and reagents

Lipofectamine 3000 was purchased from Invitrogen (Carlsbad, CA, USA). Prolong Antifade Reagent was from Molecular Probes (Invitrogen, Carlsbad, CA, USA). MEM Non-Essential Amino Acids (100×) were from Gibco (Grand Island, NY, USA). Nitrocellulose membranes were from Millipore (Bedford, MA, USA). cOmplete (EDTA-free, ROCHE). BCIP/NRT Alkaline Phosphatase Color Development Kit was from Beyotime (Shanghai, China). The primary anti-DNMT1 antibody for WB (catalogue number A16729) was from ABclonal Technology (Wuhan, China), and the one for ChIP (clone 60B1220.1; catalogue number MAB0079) was from Novus Biologicals (CO, USA).

### Human biopsies

The study protocols concerning human subjects were consistent with the principles of the Declaration of Helsinki and were approved by the clinical research ethics committee of Southern Medical University (Guangzhou, China). Femoral neck specimens were obtained from male patients with age-related osteoporosis (*n*=10) or control subjects (young men with trauma, *n*=6), recruited with written informed consent from the Department of Orthopedic Trauma, Panyu District Central Hospital of Guangzhou, China. Diagnosis of age-related osteoporosis was based on bone mineral density, measured by dual energy X-ray absorptiometry scanning (Guglielmi, 1995).

### Cell culture and siRNA transfection

C3H10T1/2 cells (ATCC, catalogue number CCL-226; American Type Culture Collection, Manassas, VA, USA) were cultured in Modified Eagle's Medium supplemented with 10% FBS, 1% non-essential amino acids, L-glutamine and sodium pyruvate (all from Gibco, Carlsbad, CA, USA). MC3T3-E1 cells (ATCC; catalogue number CRL-2594) were cultured in α-modified Eagle medium (Gibco) with 10% FBS. HEK293 cells (ATCC; catalogue number CRL-1573) were cultured in Dulbecco's Modified Eagle's Medium (Gibco) with 10% FBS. All cells were cultured at 37°C in 5% CO_2_. The siRNAs were transfected into cells using Lipofectamine 3000 according to the manufacturer's instructions. The following siRNA sequences were used: DNMT1-1, CGACTACATCAAAGGCAGCAA (5′-3′), DNMT1-2, GCAAAGAGTATGAGCCAATAT (5′-3′); NC, TTCTCCGAACGTGTCACGT (5′-3′) (GenePharma, Shanghai, China).

### Animals

Male C57BL/6 mice (3-month or 13-month-old) were purchased from the Laboratory Animal Center of Southern Medical University (Guangzhou, China). Mice were housed in plastic cages at controlled temperatures of 25±1°C, on a 12-h light:12-h dark cycle, with lights on from 06:00–18:00. Standard rodent chow and water were provided *ad libitum* throughout the study period.

### Osteogenic induction and evaluation

Cells were plated into 24-well plates and subjected to osteogenic or adipogenic differentiation induction when the cells reached 80% density or full confluence, respectively. For osteogenic induction, cells were maintained in complete medium supplemented with 50 μM L-ascorbic acid (Sigma-Aldrich), 10 mM β-glycerophosphate (Sigma-Aldrich), 10^−3^ μM dexamethasone (Sigma-Aldrich) and 1% penicillin-streptomycin (Gibco). Subsequently, the cells were stained using an ALP staining kit, according to the manufacturer's protocol (Beyotime Institute of Biotechnology, Jiangsu, China) to estimate osteogenic differentiation.

### Quantitative RT-PCR

Total RNA from C3H10T1/2 cell lines were extracted using TRIzol reagent (Life Technologies, Carlsbad, CA, USA), and reverse transcribed using HIScript QRT MIX for qPCR (+gDNA wiper) (Vazyme Biotech, Nanjing, China). The resulting cDNAs were used for PCR using the SYBR-Green Master PCR Mix (Takara Bio Inc., Shiga, Japan). All data were normalized against endogenous GAPDH controls of each sample. The primers used in the present study are listed below.

### Immunohistochemistry

Femora samples from either human or mice were decalcified for 20–30 days in decalcification solution (1.45% ETDA, 1.25% NaOH, 1.5% Glycerol, pH 7.3) at 4°C. Decalcified bones were processed and embedded in paraffin, and 5 μm sagittal-oriented sections were prepared for histological analyses. Immunohistochemistry was performed on 5 μm sections of formalin-fixed paraffin-embedded tissues. Sections were deparaffinized and dehydrated through a graded ethanol series, then the sections were repaired in citrate buffer at 60°C for 16 h, followed by 5 min in PBS, and endogenous peroxidase was blocked by incubation in 0.3% H_2_O_2_ for 15 min. After incubating with 5% BSA for 1 h, sections were incubated with appropriate primary antibodies overnight at 4°C, and then with the relevant secondary antibody for 1 h at 37°. Finally, color was developed by incubating with a DAB substrate kit (ZSGB-BIO, Beijing, China) and counterstained with hematoxylin. Immunostained sections were imaged on an Axio Scope A1 microscope (Carl Zeiss Microscopy GmbH, Jena, Germany) and processed using AxioCam HRc3 S/N 2254–ZEN 2011 software.

### Library construction and reduced representation bisulfite sequencing

A total amount of 5.2 μg genomic DNA spiked with 26 ng lambda DNA were fragmented by sonication to 200-300 bp with Covaris S220, followed by end repair and adenylation. Cytosine-methylated barcodes were ligated to sonicated DNA as per manufacturer's instructions. Then these DNA fragments were treated twice with bisulfite using EZ DNA Methylation-GoldTM Kit (Zymo Research), before the resulting single-strand DNA fragments were PCR amplificated using KAPA HiFi HotStart Uracil+ReadyMix (2X). Library concentration was quantified by Qubit^®^ 2.0 Flurometer (Life Technologies, CA, USA) and quantitative PCR, and the insert size was assayed on Agilent Bioanalyzer 2100 system. The library preparations were sequenced on an Illumina Hiseq 2500/4000 or Novaseq platform and 125 bp/150 bp paired-end reads were generated. Image analysis and base calling were performed with Illumina CASAVA pipeline, and finally 125 bp/150 bp paired-end reads were generated. After filtering the raw reads with Trimmomatic (Version 0.36), bisulfite-treated reads were aligned to the mm10 reference genome with the Bismark software (version 0.16.3). Differentially methylated regions (DMRs) were then identified using the DSS software. According to the distribution of DMRs through the genome, genes related to DMRs were defined as genes whose gene body region (from TSS to TES) or promoter region (upstream 2 kb from the TSS) have an overlap with the DMRs.

### RNA-sequencing

Total RNA was extracted from control or DNMT1 depleted C3H10T1/2 cells before or after osteoblast induction for 3 days, using TRIzol reagent (Invitrogen) following the manufacturer's protocol. Poly(A) RNA was purified from total RNA (5 μg) using poly-T oligo-attached magnetic beads, using two rounds of purification. Following purification, the mRNA was fragmented into small pieces using divalent cations under elevated temperature. The cleaved RNA fragments were then reverse transcribed to create the final cDNA library, in accordance with the protocol for the TruSeq RNA Sample Preparation v.2 (catalogue numbers RS-122-2001 and RS-122-2002) (Illumina). The paired-end sequencing was carried out on an Illumina Hiseq 2500 following the manufacturer's recommended protocol. Before assembly, the low-quality reads (defined as reads containing sequencing adaptors; reads containing sequencing primer; and nucleotides with a q quality score lower than 20) were removed using the Trimmomatic software. Sequencing reads were aligned to the reference genome using HISAT2 package. The mapped reads of each sample were assembled and counted using the featureCounts package. After a matrix of read counts was generated, differential gene expression was analysed using the R package edgeR. The differentially expressed genes were selected by R package with log2 (fold change) values of ≥1 or log2 (fold change) values of ≤−1 and with statistical significance of *P*<0.05.

### ChIP-qPCR

ChIP-qPCR assays were performed on chromatin isolated from ∼4×10^6^ cells with the ChIP Assay Kit (catalogue number 9005 s, CST, USA) according to the manufacturer's instructions.

The following primers were used: RORA F, CTCTCGCCCGTCTCCTTTTC; RORA R CTGCCCGGTTCGCTGG; Fgfr2 F, TCAAAGGAACGCGCCCAGTAG; Fgfr2 R, GAAGGCGCGGGTAAACCTATTT.

### iTRAQ labeling and LC–MS/MS proteomics

Articular cartilages were removed and bone marrow were flushed out with PBS. Total protein samples were then extracted from the distal femur of the 3-month or 16-month C57BL/6 mice with lysis buffer and centrifuged at 12,000 ***g*** for 30 min at 4°C. At the end, 100 μg of each protein was used for enzymolysis and iTRAQ^®^ labeling. iTRAQ^®^ labeling was performed using a previously described method (Liu et al., 2010) and the peptide samples were labeled with the iTRAQ^®^ Reagent Multiplex Kit (Applied Biosystems, Foster City, CA, USA). LC–MS/MS was performed by the Fitgene Biological Technology Co. Ltd (Fitgene, Guangzhou, China). Protein Pilot software v4.0 (Applied Biosystems) was used to convert the raw data into peak lists. The average relative expression, *P*-values, error factors, lower confidence interval, and upper confidence interval were calculated with Protein Pilot and then exported into Excel.

### Bioinformatics analysis

The transcriptomes of C310T1/2 cells, before or after osteoblastic induction, were subjected to gene ontology (GO) and KEGG pathway analysis using the ClusterProfiler and enrichplot Bioconductor R packages (Yu et al., 2012), following website guidelines. The cnetplot function provided in the ClusterProfiler package was used to visualize GO functional enrichment results. For reduced representation bisulfite sequencing, the vioplot R package (https://cran.r-project.org/web/packages/vioplot/index.html) was used to draw violin plots to rank differentially methylated regions.

### Statistical analysis

All statistical analyses were conducted using GraphPad Prism 5 software. Data were analyzed using two-tailed *t*-tests or one-way analysis of variance with multiple comparisons, followed by the Bonferroni post-hoc test for significance. A *P*-value less than 0.05 was considered statistically significant.

## Supplementary Material

Supplementary information

## References

[BIO058534C1] Akiyama, H. and Lefebvre, V. (2011). Unraveling the transcriptional regulatory machinery in chondrogenesis. *J. Bone Miner. Metab.* 29, 390-395. 10.1007/s00774-011-0273-9 21594584 PMC3354916

[BIO058534C2] Amato, R. J., Stephenson, J., Hotte, S., Nemunaitis, J., Belanger, K., Reid, G. and Martell, R. E. (2012). MG98, a second-generation DNMT1 inhibitor, in the treatment of advanced renal cell carcinoma. *Cancer Invest.* 30, 415-421. 10.3109/07357907.2012.675381 22571342

[BIO058534C3] Bestor, T. H. (2000). The DNA methyltransferases of mammals. *Hum. Mol. Genet.* 9, 2395-2402. 10.1093/hmg/9.16.2395 11005794

[BIO058534C4] Bowers, R. R., Kim, J. W., Otto, T. C. and Lane, M. D. (2006). Stable stem cell commitment to the adipocyte lineage by inhibition of DNA methylation: role of the BMP-4 gene. *Proc. Natl. Acad. Sci. USA* 103, 13022-13027. 10.1073/pnas.0605789103 16916928 PMC1559746

[BIO058534C5] Chatterjee, T. K., Idelman, G., Blanco, V., Blomkalns, A. L., Piegore, Jr., M. G., Weintraub, D. S., Kumar, S., Rajsheker, S., Manka, D., Rudich, S. M. et al. (2011). Histone deacetylase 9 is a negative regulator of adipogenic differentiation. *J. Biol. Chem.* 286, 27836-27847. 10.1074/jbc.M111.262964 21680747 PMC3149373

[BIO058534C6] Chen, Q., Shou, P., Zheng, C., Jiang, M., Cao, G., Yang, Q., Cao, J., Xie, N., Velletri, T., Zhang, X. et al. (2016). Fate decision of mesenchymal stem cells: adipocytes or osteoblasts? *Cell Death Differ* 23, 1128-1139. 10.1038/cdd.2015.168. 26868907 PMC4946886

[BIO058534C7] Elliott, E. N., Sheaffer, K. L., Schug, J., Stappenbeck, T. S. and Kaestner, K. H. (2015). Dnmt1 is essential to maintain progenitors in the perinatal intestinal epithelium. *Development* 142, 2163-2172. 10.1242/dev.117341 26023099 PMC4483766

[BIO058534C8] Espada, J. (2012). Non-catalytic functions of DNMT1. *Epigenetics* 7, 115-118. 10.4161/epi.7.2.18756 22395459

[BIO058534C9] Fernandez-Moure, J. S., Corradetti, B., Chan, P., Van Eps, J. L., Janecek, T., Rameshwar, P., Weiner, B. K. and Tasciotti, E. (2015). Enhanced osteogenic potential of mesenchymal stem cells from cortical bone: a comparative analysis. *Stem Cell Res Ther* 6, 203. 10.1186/s13287-015-0193-z 26503337 PMC4620594

[BIO058534C10] Furst, R. W., Kliem, H., Meyer, H. H. and Ulbrich, S. E. (2012). A differentially methylated single CpG-site is correlated with estrogen receptor alpha transcription. *J. Steroid Biochem. Mol. Biol.* 130, 96-104. 10.1016/j.jsbmb.2012.01.009 22342840

[BIO058534C11] Georgia, S., Kanji, M. and Bhushan, A. (2013). DNMT1 represses p53 to maintain progenitor cell survival during pancreatic organogenesis. *Genes Dev.* 27, 372-377. 10.1101/gad.207001.112 23431054 PMC3589554

[BIO058534C12] Goll, M. G. and Bestor, T. H. (2005). Eukaryotic cytosine methyltransferases. *Annu. Rev. Biochem.* 74, 481-514. 10.1146/annurev.biochem.74.010904.153721 15952895

[BIO058534C13] Gordon, J. A., Hassan, M. Q., Saini, S., Montecino, M., van Wijnen, A. J., Stein, G. S., Stein, J. L. and Lian, J. B. (2010). Pbx1 represses osteoblastogenesis by blocking Hoxa10-mediated recruitment of chromatin remodeling factors. *Mol. Cell. Biol.* 30, 3531-3541. 10.1128/MCB.00889-09 20439491 PMC2897555

[BIO058534C14] Guglielmi, G. (1995). Quantitative computed tomography (QCT) and dual X-ray absorptiometry (DXA) in the diagnosis of osteoporosis. *Eur. J. Radiol.* 20, 185-187. 10.1016/0720-048X(95)00647-9 8536745

[BIO058534C15] Kallenberger, L., Erb, R., Kralickova, L., Patrignani, A., Stockli, E. and Jiricny, J. (2019). Ectopic Methylation of a Single Persistently Unmethylated CpG in the Promoter of the Vitellogenin Gene Abolishes Its Inducibility by Estrogen through Attenuation of Upstream Stimulating Factor Binding. *Mol. Cell. Biol.* 39, 00436-19. 10.1128/MCB.00436-19 PMC6851352 31548262

[BIO058534C16] Katagiri, T., Yamaguchi, A., Ikeda, T., Yoshiki, S., Wozney, J. M., Rosen, V., Wang, E. A., Tanaka, H., Omura, S. and Suda, T. (1990). The non-osteogenic mouse pluripotent cell line, C3H10T1/2, is induced to differentiate into osteoblastic cells by recombinant human bone morphogenetic protein-2. *Biochem. Biophys. Res. Commun.* 172, 295-299. 10.1016/S0006-291X(05)80208-6 1699539

[BIO058534C17] Kawakami, Y., Rodriguez-Leon, J. and Izpisua Belmonte, J. C. (2006). The role of TGFbetas and Sox9 during limb chondrogenesis. *Curr. Opin. Cell Biol.* 18, 723-729. 10.1016/j.ceb.2006.10.007 17049221

[BIO058534C18] Li, E., Bestor, T. H. and Jaenisch, R. (1992). Targeted mutation of the DNA methyltransferase gene results in embryonic lethality. *Cell* 69, 915-926. 10.1016/0092-8674(92)90611-F 1606615

[BIO058534C19] Li, B., Zhao, J., Ma, J. X., Li, G. M., Zhang, Y., Xing, G. S., Liu, J. and Ma, X. L. (2018). Overexpression of DNMT1 leads to hypermethylation of H19 promoter and inhibition of Erk signaling pathway in disuse osteoporosis. *Bone* 111, 82-91. 10.1016/j.bone.2018.03.017 29555308

[BIO058534C20] Liberti, D. C., Zepp, J. A., Bartoni, C. A., Liberti, K. H., Zhou, S., Lu, M., Morley, M. P. and Morrisey, E. E. (2019). Dnmt1 is required for proximal-distal patterning of the lung endoderm and for restraining alveolar type 2 cell fate. *Dev. Biol.* 454, 108-117. 10.1016/j.ydbio.2019.06.019 31242446 PMC6822389

[BIO058534C21] Lim, K.-H., Park, E.-S., Kim, D. H., Cho, K. C., Kim, K. P., Park, Y. K., Ahn, S. H., Park, S. H., Kim, K.-H., Kim, C. W. et al. (2018). Suppression of interferon-mediated anti-HBV response by single CpG methylation in the 5'-UTR of TRIM22. *Gut* 67, 166-178. 10.1136/gutjnl-2016-312742 28341749

[BIO058534C22] Liu, Y., Wu, J., Yan, G., Hou, R., Zhuang, D., Chen, L., Pang, Q. and Zhu, J. (2010). Proteomic analysis of prolactinoma cells by immuno-laser capture microdissection combined with online two-dimensional nano-scale liquid chromatography/mass spectrometry. *Proteome Sci* 8, 2. 10.1186/1477-5956-8-2 20205839 PMC2825229

[BIO058534C23] Liu, R., Kim, K. Y., Jung, Y. W. and Park, I. H. (2016). Dnmt1 regulates the myogenic lineage specification of muscle stem cells. *Sci. Rep.* 6, 35355. 10.1038/srep35355 27752090 PMC5082760

[BIO058534C24] Nagarajan, A., Roden, C. and Wajapeyee, N. (2014). Reduced representation bisulfite sequencing to identify global alteration of DNA methylation. *Methods Mol. Biol.* 1176, 23-31. 10.1007/978-1-4939-0992-6_3 25030916

[BIO058534C25] Pierce, J. L., Begun, D. L., Westendorf, J. J. and McGee-Lawrence, M. E. (2019). Defining osteoblast and adipocyte lineages in the bone marrow. *Bone* 118, 2-7. 10.1016/j.bone.2018.05.019 29782940 PMC6240509

[BIO058534C26] Schubeler, D. (2015). Function and information content of DNA methylation. *Nature* 517, 321-326. 10.1038/nature14192 25592537

[BIO058534C27] Sheaffer, K. L., Kim, R., Aoki, R., Elliott, E. N., Schug, J., Burger, L., Schubeler, D. and Kaestner, K. H. (2014). DNA methylation is required for the control of stem cell differentiation in the small intestine. *Genes Dev.* 28, 652-664. 10.1101/gad.230318.113 24637118 PMC3967052

[BIO058534C28] Sottile, V., Halleux, C., Bassilana, F., Keller, H. and Seuwen, K. (2002). Stem cell characteristics of human trabecular bone-derived cells. *Bone* 30, 699-704. 10.1016/S8756-3282(02)00674-9 11996907

[BIO058534C29] Taylor, S. E. B., Li, Y. H., Smeriglio, P., Rath, M., Wong, W. H. and Bhutani, N. (2016). Stable 5-Hydroxymethylcytosine (5hmC) Acquisition Marks Gene Activation During Chondrogenic Differentiation. *J. Bone Miner. Res.* 31, 524-534. 10.1002/jbmr.2711 26363184 PMC4860191

[BIO058534C30] Wang, W., Strecker, S., Liu, Y., Wang, L., Assanah, F., Smith, S. and Maye, P. (2015). Connective Tissue Growth Factor reporter mice label a subpopulation of mesenchymal progenitor cells that reside in the trabecular bone region. *Bone* 71, 76-88. 10.1016/j.bone.2014.10.005 25464947 PMC4274218

[BIO058534C31] Wong, K. K. (2020a). DNMT1 as a therapeutic target in pancreatic cancer: mechanisms and clinical implications. *Cell Oncol (Dordr)* 43, 779-792. 10.1007/s13402-020-00526-4 32504382 PMC12990712

[BIO058534C32] Wong, K. K. (2020b). DNMT1: A key drug target in triple-negative breast cancer. *Semin. Cancer Biol* 72, 198-213. 10.1016/j.semcancer.2020.05.010 32461152

[BIO058534C33] Yamashita, M., Inoue, K., Saeki, N., Ideta-Otsuka, M., Yanagihara, Y., Sawada, Y., Sakakibara, I., Lee, J., Ichikawa, K., Kamei, Y. et al. (2018). Uhrf1 is indispensable for normal limb growth by regulating chondrocyte differentiation through specific gene expression. *Development* 145, pii: dev.157412. 10.1242/dev.157412 29180567

[BIO058534C34] Yu, G., Wang, L.-G., Han, Y. and He, Q.-Y. (2012). clusterProfiler: an R package for comparing biological themes among gene clusters. *OMICS* 16, 284-287. 10.1089/omi.2011.0118 22455463 PMC3339379

[BIO058534C35] Zhang, M., Lu, Q., Miller, A. H., Barnthouse, N. C. and Wang, J. (2016). Dynamic epigenetic mechanisms regulate age-dependent SOX9 expression in mouse articular cartilage. *Int. J. Biochem. Cell Biol.* 72, 125-134. 10.1016/j.biocel.2016.01.013 26806292 PMC4762732

