# Supplementary material for: DNMT1 is a negative regulator of osteogenesis
Source: Biol Open. 2022 Mar 3;11(3):bio058534. doi: 10.1242/bio.058534 (PMC8905718; doi:10.1242/bio.058534)
Supplement: Supplementary information [file biolopen-11-058534-s1.pdf]

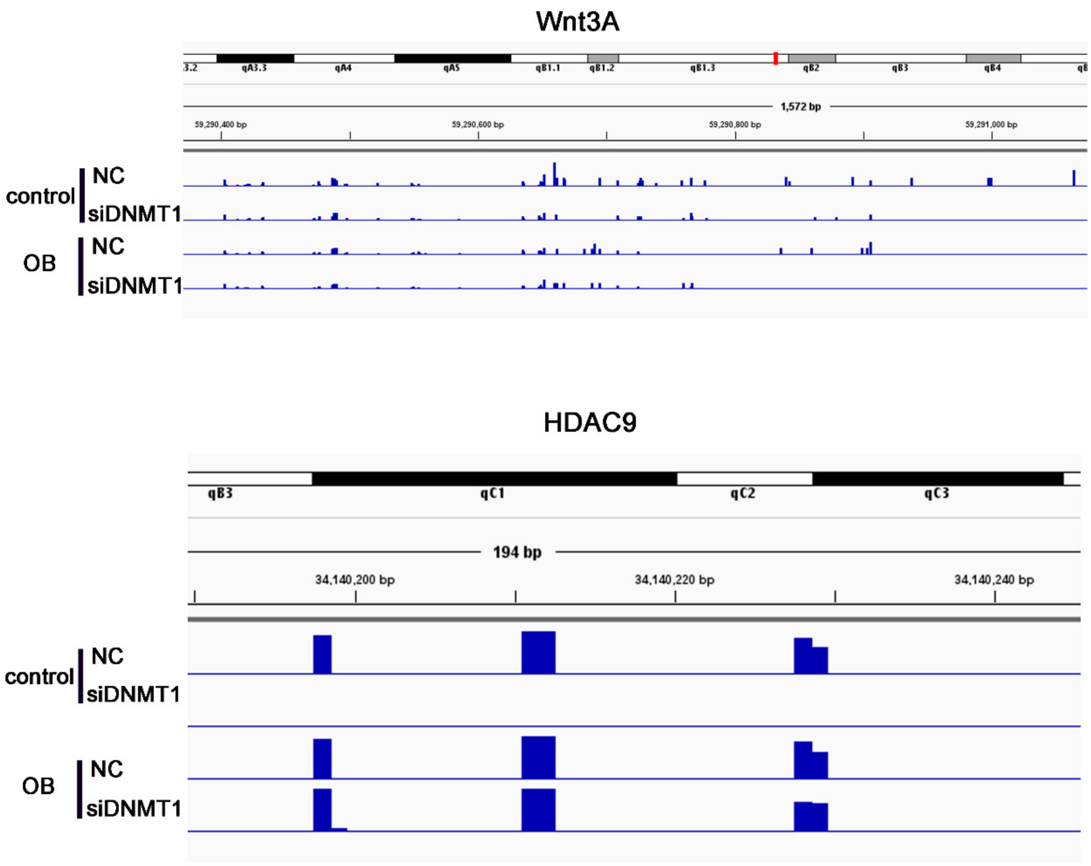

Fig. S1. Two hypomethylated loci in DNMT1 depleted MSCs.

C3H10T1/2 mesenchymal stem cells (MSCs) were transfected with DNMT1 (or non-targeting, NC) siRNA, induced for early osteoblast (OB) differentiation, and analyzed for global DNA methylation by reduced representation bisulfite sequencing. The methylation levels of 2 individual loci at the promoter of Wnt3a, or an intron of HDAC9, were viewed by IGV, respectively.

Biology Open • Supplementary information
